# A New Index for the Prediction of 30-Day Mortality in Patients With Pulmonary Embolism: The Pulmonary Embolism Mortality Score (PEMS)

**DOI:** 10.1177/0003319721993346

**Published:** 2021-02-09

**Authors:** Alexey Surov, Mideia Akritidou, Andreas Gunther Bach, Nikolaos Bailis, Marianne Lerche, Hans Jonas Meyer, Maciej Pech, Andreas Wienke

**Affiliations:** 1 Department of Radiology, University of Leipzig, Germany; 2 Department of Radiology and Nuclear Medicine, Otto-von-Guericke University Magdeburg, Germany; 3 Department of Internal Medicine, Otto-von-Guericke University Magdeburg, Germany; 4 Department of Radiology, Martin-Luther-University Halle-Wittenberg, Halle, Germany; 5 Department of Respiratory Medicine, University of Leipzig, Germany; 6 Institute of Medical Epidemiology, Biostatistics, and Informatics, Martin-Luther-University Halle-Wittenberg, Halle, Germany

**Keywords:** acute pulmonary embolism, 30-day mortality, simplified pulmonary embolism index, computer tomographic pulmonary angiography

## Abstract

Our aim was to analyze possibility of combination of basic clinical and radiological signs to predict 30-day mortality after acute pulmonary embolism (PE). We included 486 patients. Age, gender, simplified pulmonary embolism index (sPESI), pH, troponin, N-terminal natriuretic peptide, minimal systolic and diastolic blood pressure, O_2_ saturation, syncope, need for vasopressors, thrombotic obstruction, vessel diameter, short axis ratio right ventricle/left ventricle, and contrast medium reflux into the inferior vena cava (IVC) were analyzed. A backward algorithm in a logistic regression model was used to identify relevant risk factors. Multiple logistic regression analysis identified that sPESI, pH, minimal diastolic blood pressure, IVC reflux, and need for vasopressors influenced 30-day mortality. A score for mortality prediction was constructed (the Pulmonary Embolism Mortality Score): sPESI >2 points (1 point), pH <7.35 (1 point), minimal diastolic blood pressure <45 mm Hg (1 point), IVC reflux (1 point), and need for vasopressors (2 points). Patients with >3 points showed higher 30-day mortality (sensitivity: 84.9%, specificity: 83.0%, positive predictive value: 51.8%, negative predictive value: 96.2%). The net reclassification improvement compared with the sPESI was 0.94 (95% CI = 0.73-1.15). In conclusion, a new score can predict 30-day mortality in patients with PE and is more sensitive than sPESI.

## Introduction

Acute pulmonary embolism (PE) is associated with a high mortality.^1,2^ In fact, some authors reported a mortality rate of up to 18%.^3^ A large meta-analysis reported a calculated mortality of 10.7%.^4^ Thus, an immediate risk stratification of patients with acute PE at the time of presentation is very important. Currently, there are several scores to predict clinical outcome in patients with PE.^5–7^ Most of them are based on clinical and serological parameters.

In daily clinical practice, the severity of acute PE can be estimated by using the simplified pulmonary embolism index (sPESI). This score is based on clinical parameters and includes 6 equally weighted variables as follows: age >80 years, presence of cancer, chronic heart failure or chronic pulmonary disease, systolic blood pressure <100 mm Hg, and arterial oxyhemoglobin saturation <90%.^5,8^ According to the literature, sPESI shows good interobserver variability^9^ and can accurately discriminate patients with low risk of mortality (0 points).^10^

However, some authors suggested that the sPESI might ignore a significant proportion of intermediate risk patients. For example, Cordeanu et al mentioned that 34% of the patients with a sPESI of 0 had elevated cardiac biomarkers or right ventricular dysfunction (RVD) or both.^11^ Right ventricular dysfunction and/or elevation of cardiac biomarkers, liketroponin, significantly increases the risk of mortality.^12,13^

Computer tomographic pulmonary angiography (CTPA) is the current gold standard for the diagnosis of PE. Furthermore, several studies suggested that different CTPA parameters can be used as predictors of morbidity/mortality in patients with PE.^12,14–16^

Presumably, the combination of clinical, radiological, and serological parameters may improve risk stratification of patients with acute PE. The purpose of the present study was to evaluate the combination of basic clinical and radiological signs as predictors of 30-day mortality in PE based on a large cohort.

## Materials and Methods

This retrospective study included data from 2 centers (Martin-Luther University Halle-Wittenberg and University of Leipzig), and it was approved by the institutional review boards.

For the study, the electronic databases of the radiological departments were screened and all patients with acute PE were extracted. There were 612 patients. Inclusion criteria for the study were available clinical data (age and gender, sPESI, minimal systolic and diastolic blood pressures, heart rate, episodes of syncope, and need for vasopressors), available CTPA images in PACS (picture archiving and communication system), and available biochemical data including pH, O_2_ saturation, troponin, and N-terminal natriuretic peptide level (optional). Patients with incomplete data were excluded. Furthermore, patients with anamnestic known chronic PE were excluded. Patients with known terminal malignancies were also excluded. Overall, 126 patients were excluded from the study. Therefore, our sample comprised 486 patients with PE, 240 (49.4%) females and 246 (50.6%) males, mean age, 63.8 ± 16.1 years and median age, 66 years. The following clinical and serological parameters were analyzed: age and gender of the patients, mortality within the observation time of 30 days, simplified PESI score, pH troponin level (pg/mL), and N-terminal natriuretic peptide (BNP, pg/mL) level, minimal systolic and diastolic blood pressures (mm Hg), heart rate, O_2_ saturation, episodes of syncope, and need for vasopressors.

In all cases, the diagnosis of PE was confirmed by CTPA. Computer tomographic pulmonary angiography was performed on diverse multislice CT scanners (Ingenuity 128, Philips; Somatom Sensation 64, Siemens; Aquillon 64, Toshiba). In all cases intravenous administration of an iodinated contrast agent (60 mL Imeron 400 MCT, Bracco Imaging Germany GmbH) was given at a rate of 3.0 to 4.0 mL/s via a peripheral venous line. Automatic bolus tracking was performed in the pulmonary trunk with a trigger of 100 Hounsfield units. Typical imaging parameters were 100 to 120 kVp, 125 to 300 mAs, slice thickness 1 mm, and a pitch of 0.6 to 1.2.

**Figure 1.**
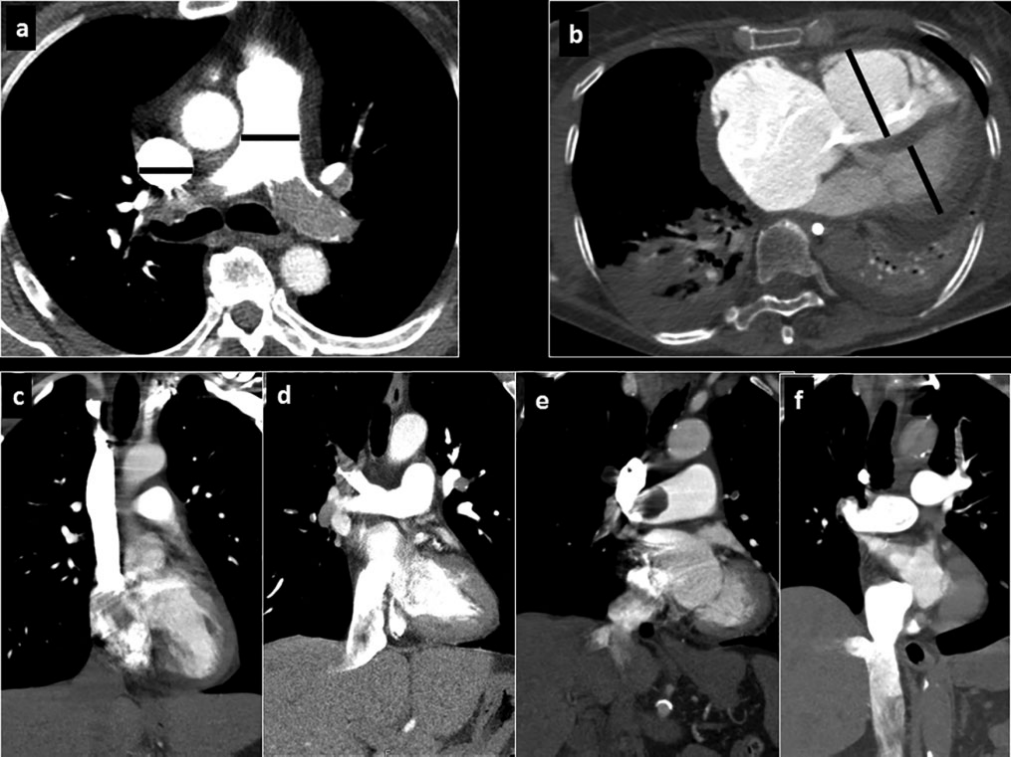
Measurements on CTPA performed for the present study: A, Diameter of the pulmonary trunk and superior cava vein; (B) RV/LV diameter ratio and ventricular septal bowing. C, None reflux into the inferior cava vein. D, Grade 1: reflux into the suprahepatic IVC only. E, Grade 2: reflux into the intrahepatic IVC as well and into the hepatic veins. F, Grade 3: infrahepatic reflux. CTPA indicates computer tomographic pulmonary angiography; IVC, inferior vena cava; RV/LV, right ventricle/left ventricle.

For the present study, the following radiological parameters were measured: thrombotic obstruction index, diameter of the pulmonary trunk (mm), short axis ratio right ventricle/left ventricle (RV/LV), diameter of the superior cava vein (mm), and reflux of contrast medium into the inferior vena cava (IVC).^13^ Every vessel diameter was regarded as the largest distance from wall to wall on axial slices (Figure 1). Right and left ventricular diameters were estimated at the largest points between the inner margins of the interventricular septum and the ventricle wall.

Reflux into the IVC was estimated on axial and coronal images and was quantified in a 4-point scale^13^: no reflux (0 points), subcardial reflux into IVC (1 point), intrahepatic reflux in IVC (2 points), and subhepatic reflux in IVC (Figure 1C-F).

**Figure 2.**
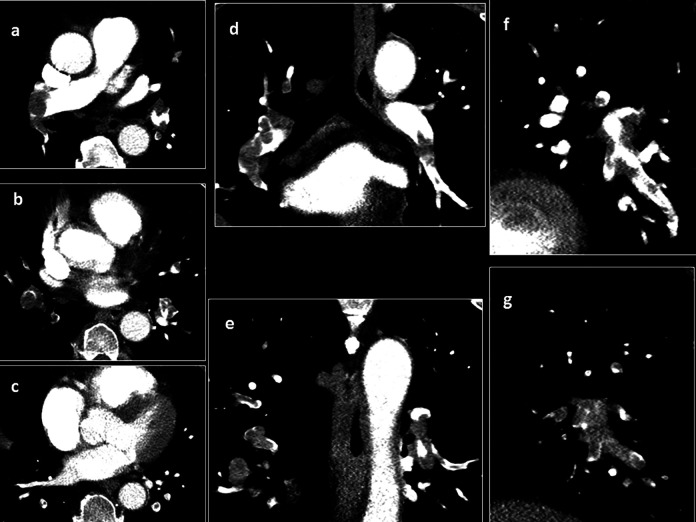
Computer tomographic pulmonary angiography (CTPA) images (axial, A-C; coronal, D, E; sagittal, F, G) for estimation of the thrombotic obstruction index (mastora score).

Thrombotic obstruction index of the pulmonary arteries was calculated according to Mastora et al (Mastora score).^17^ For this index, the obstruction of the mediastinal, lobar, and segmental arteries was quantified by a percentage or ratio (thrombotic occluded lumen divided by the total vessel lumen) × 100%. The analysis of thrombotic obstruction was performed in 3 dimensional images (Figure 2). Furthermore, the sum of the percentages of all arteries was calculated as the global obstruction score.

All images were available in digital format and were analyzed on PACS workstations (Singo Plaza, Siemens Healthineers and Centricity PACS, GE Medical Systems). All images were reanalyzed for this study by 2 radiologists with 5 and 4 years of radiological experience. If there was any disagreement, a senior radiologist (17 years’ experience) helped resolve these differences.

### Statistical Analysis

Statistical analysis was performed using the SPSS package (IBM SPSS Statistics for Windows, version 225.0: IBM corporation). The collected data were evaluated by means of descriptive statistics (absolute and relative frequencies). A backward algorithm in a logistic regression model was used to identify relevant risk factors of 30-day mortality. To quantify the advantage of the new score compared with the sPESI, the net reclassification improvement (NRI) was calculated with 95% CI.^18,19^ The Mantel-Haenzsel test was used to test for trend.

## Results

Of the 486 patients, hemodynamically unstable PE was diagnosed in 76 (15.6%) patients and hemodynamically stable PE in 410 (84.4%) patients.

Overall, 86 (17.7%) patients died and 400 patients survived within the 30-day observation time. Use of the sPESI (≥2 points) for prediction of 30-day mortality yielded a sensitivity of 83.7%, a specificity of 45.2%, a positive predictive value of 24.7%, a negative predictive value of 92.8%, and an accuracy of 52.1%.

The next step was multiple logistic regression analysis of the acquired clinical, chemical, and radiological data were performed. It identified that the sPESI score, pH value, minimal diastolic blood pressure, IVC reflux, and need for vasopressors were associated with 30-day mortality (Table 1). Other analyzed variables did not influence the 30-day mortality.

**Table 1.** Multiple Logistic Regression Analysis of the Acquired Parameters for Prediction of 30-Day Mortality in Patients With Acute Pulmonary Embolism.

Parameters	Odds ratio	95% CI	*P*
**sPESI**	1.36	1.04-1.76	.023
**pH**	0.07	0.01-0.46	.010
**Minimal diastolic blood pressure (mm Hg)**	0.97	0.96-0.99	.008
**Need for vasopressors**	9.41	4.78-18.51	<.001
**IVC reflux grade 3**	2.71	1.44-5.10	.002

Abbreviations: IVC, inferior vena cava; sPESI, simplified pulmonary embolism index.

Furthermore, a score for prediction of mortality in PE (Pulmonary Embolism Mortality Score, PEMS) based on sensitivity/specificity of the 5 variables was constructed as follows: sPESI ≥2 points (1 point), pH value under 7.35 (1 point), minimal diastolic blood pressure <45 mm Hg (1 point), IVC reflux grade 3 (1 point), and need for vasopressors (2 points).

Also, frequency of mortality events for every point on the new score were analyzed (Table 2). Patients with 3 and more points showed a high mortality within the observation time of 30 days ranging from 37.0% (3 points) to 88.9% (6 points; *P* < .001). Threshold of 3 points yielded a sensitivity of 84.9%, a specificity of 83.0%, a positive predictive value of 51.8%, a negative predictive value of 96.2%, and an accuracy of 83.3%.

**Table 2.** Thirty-Day Mortality Rate in Dependence on Points on the New Score (PEMS).

Points on PEMS	Deaths, all patients	Deaths, patients with hemodynamically stable PE	Deaths, patients with hemodynamically unstable PE	Patients, total
**0**	1 (0.7%)	1 (0.7%)	0	141
**1**	8 (5.3%)	8 (5.6%)	0	150
**2**	4 (7.4%)	4 (8.9%)	0	54
**3**	20 (37.0%)	14 (38.9%)	6 (33.3%)	54
**4**	23 (45.1%)	14 (43.8%)	9 (47.4%)	51
**5**	22 (81.5%)	12 (92.3%)	10 (71.4%)	27
**6**	8 (88.9%)	0	8 (88.9%)	9
**Total**	86	53	33	486
***P* value for trend**	<.001	<.001	<.001	

Abbreviations: PE, pulmonary embolism; PEMS, Pulmonary Embolism Mortality Score.

**Figure 3.**
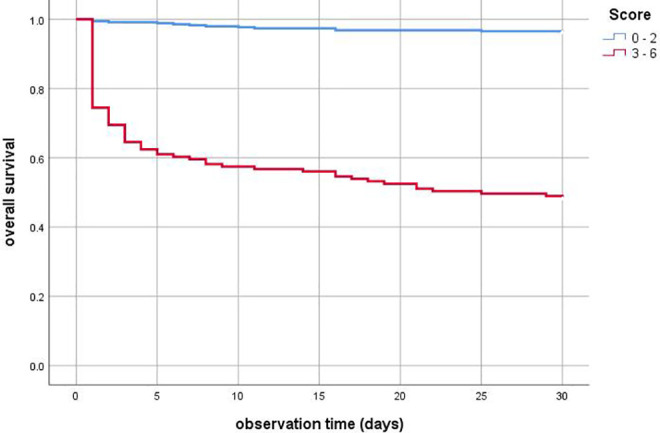
Kaplan-Meier curves of patients with different PEMS (pulmonary embolism mortality score) values. Patients with ≥3 points on the PEMS had shorter survival within the overall observation time than patients with 0 to 2 points (*P* < .001).

Finally, the Kaplan-Meier curves demonstrated that patients with ≥3 points on the PEMS had shorter survival within the overall observation time than patients with 0 to 2 points (Figure 3).

Reclassification of patients with and without events was also carried out. For 57 patients who died during 30 days, the reclassification improved using the new score, and for 13 patients it became worse (of 86). The net gain in reclassification proportion for patients who did not die 64 individuals were reclassified down and 235 were reclassified up (of 400). Therefore, the NRI was estimated as 0.94 (95% CI: 0.73-1.15) showing a considerable improvement with the new score compared with the sPESI score.

Furthermore, a subanalysis of the proposed score for prediction of 30-day mortality was performed for the subgroups with hemodynamically unstable (n = 76) and hemodynamically stable PE (n = 410). In the subgroup with hemodynamically unstable PE, PEMS had a sensitivity of 100.0%, a specificity of 37.2%, a positive predictive value of 55.0%, a negative predictive value of 100.0%, and an accuracy of 64.5%.

In the subgroup with hemodynamically stable PE, PEMS yielded a sensitivity of 75.5%, a specificity of 88.5%, a positive predictive value of 49.4%, a negative predictive value of 96.0%, and an accuracy of 86.8%.

## Discussion

According to the literature, several clinical, chemical, and CT signs can predict outcome in PE.^5–7,12,13^ Also, several scores as combination of these factors were proposed. Frequently, the sPESI is used, which was created for the risk stratification of PE patients.^5–7^ This score is widely respected in clinical practice because its simplicity and high sensitivity.^5,8–10^ However, some authors noted several limitations of the sPESI. Firstly, the sPESI has a high sensitivity but a low specificity.^20^ So far, Lankeit et al showed that sPESI had 94% sensitivity and 40% specificity as predictor of 30-day mortality in patients with PE.^21^ Our data are in agreement with those of the literature.^20,21^ Furthermore, the sPESI is based on clinical signs only. The score does not consider relevant radiological and/or echocardiographic parameters. Also, relevant cardiac biomarkers are not included into the score. This can be a serious limitation. In fact, it has been shown that about 30% of patients with a low risk (0 points on sPESI) had a RVD and/or an elevation of cardiac biomarkers.^9^ According to the literature, elevated troponin level is associated with RVD in PE.^22^ Additionally, it has been reported that troponin level is an independent predictor of short-term outcome in patients with PE.^14,22,23^ Also, BNP can predict clinical outcome in patients with PE.^24^

Patient gender also plays a role in PE. Barrios et al found that female gender was an independent predictor of all-cause (adjusted OR: 1.56; 95% CI: 1.07-2.28; *P* = .02) and PE-specific mortality (adjusted OR: 1.85; 95% CI: 1.02-3.33; *P* = .04).^25^ Similarly, Agarwal et al also reported that compared with men, women had a significantly higher in-hospital mortality after admission with acute PE.^26^ Finally, a different predictive accuracy of the sPESI among gender was reported.^27^ The predictive ability of the sPESI score as prognosticator of all cause in-hospital mortality was higher in females compared with males.^27^ Our data did not confirm these results; age and gender did not influence the 30-day mortality in the present study.

According to the literature, some clinical signs such as syncope can also be used as predictor of PE outcome.^28,29^ Roncon et al showed that PE patients with syncope/presyncope had a higher 30-day all-cause mortality.^28^ It is well known that PE patients with shock and need for vasopressors represent a group with very high mortality.^29^

Some CTPA parameters were also predictors of PE morbidity/mortality.^14–16^ Computer tomographic pulmonary angiography signs of RVD have been shown as very important predictors.^12,14–16,28^ A large meta-analysis including 4661 patients that CT-detected right ventricle dilation calculated as a ratio RV/LV was associated with an increased 30-day mortality.^16^ Inferior vena cava reflux is another important CTPA parameter. It has been shown that IVC reflux correlated with tricuspid regurgitation and with the level of troponin and N-terminal natriuretic peptide.^30,31^ Also, patients with substantial IVC reflux had higher mortality rate within 30 days compared with patients with no or minimal reflux.^15,32^

The European Society of Cardiology proposed in 2014 a risk stratification strategy for patients with acute PE.^33^ The PESI or sPESI categorizes hemodynamically stable patients into “low-risk” and “intermediate-risk” categories. Right ventricular dysfunction and troponin levels should be used for further categorization of intermediate-risk patients into 2 groups as follows: “intermediate-high-risk” and “intermediate-low-risk.” Patients with the intermediate-high-risk show RVD and increased troponin.^33^ In contrast, patients with an intermediate-low-risk have either RVD or elevated troponin or with both absent. However, as reported by some authors, the risk stratification for death by the sPESI showed deficits.^34^ So far, it has been shown that death rate was 22% in “high-risk” (95% CI: 14.0-29.8), 7.7% in “intermediate-high-risk” (95% CI: 4.5-10.9), and 6.0% in “intermediate-low-risk” patients (95% CI: 3.4-8.6).^34^

Lankeit et al studied the prognostic role of heart-type fatty acid-binding protein (H-FABP) in acute PE and found that this protein may predict 30-day mortality in PE.^35^ Furthermore, the authors conducted a score as follows: positive H-FABP bedside-test “weighted” 1.5 points; tachycardia, 2.0 points; and syncope, 1.5 points. By receiver operating characteristic curve analysis, the optimal cutoff value of 3.0 points was proposed for discriminating between patients with an adverse 30-day outcome and those with a favourable course (AUC, 0.847 [0.746-0.949]).^35^ This score was obtained based on a small sample of 136 normotensive patients with PE. Heart-type fatty acid-binding protein cannot be routinely estimated in every emergency department. Furthermore, the score did not contain radiological signs of RVD.

Some authors attempted to construct new scores of PE based on CTPA findings and clinical values. Bova et al constructed a score (Bova score) including both clinical and radiological features.^36^ This score is composed of 4 variables: systolic blood pressure 90 to 100 mm Hg (2 points), heart rate ≥110 beats/min (1 point), troponin elevation (2 points), and echocardiographic or computer tomography pulmonary angiography right heart dysfunction (2 points).^36^ The Bova score ranges from 0 points (all variables absent) to 7 points (all variables present). The score categorizes 3 risk classes at low (≤2 points), intermediate (3-4 points), and high (≥5 points) risk of PE-related complications, defined as death from PE, hemodynamic collapse, or recurrent nonfatal PE.^36^ This score was also validated on independent cohorts. Patients with a Bova risk score ≤2 had in-hospital 3.7% and 30-day 4%, patients with a Bova risk score 3 to 4 had in-hospital 15% and 30-day 18% and patients with a Bova risk score ≥5 had in-hospital 37% and 30-day 42% of PE-related complications, respectively.^37^ Furthermore, the Bova score showed an AUC of 0.74 (95% CI: 0.68-0.80) for the main endpoint in the validation cohort.^37^ However, some authors indicated that the Bova score failed to identify patients at highest risk.^38^

We hypothesize that adding signs of cardiovascular failure may improve the potential of the established clinical scores like the sPESI. Therefore, we included into the present analysis, in addition to the sPESI values, troponin, BNP and pH values, gender, and hemodynamic parameters such as syncope, minimal systolic and diastolic blood pressure, need for vasopressors, and different CTPA parameters. The present results confirmed our hypothesis. Furthermore, the present work identified other interesting aspects. Surprisingly, multivariate logistic regression analysis showed that oxygen saturation, heart rate, syncope, systolic blood pressure, troponin, and BNP levels were not associated with 30-day mortality in PE. Only the sPESI, pH, minimal diastolic blood pressure, need for vasopressors, and contrast medium reflux into the IVC on CTPA were linked to 30-day mortality. Interestingly, the need for vasopressors and contrast medium reflux into the inferior cava vein on CTPA had higher odds ratios for prediction of 30-day mortality than the sPESI. Therefore, these parameters are more sensitive as a mortality predictor in PE, and therefore, they should be included into a risk calculation. As shown, the new score has acceptable sensitivity and specificity, namely 84.9% and 83.0%, respectively. Our score includes basic clinical and radiological parameters. It can immediately stratify risk in PE. Furthermore, PEMS is more sensitive than sPESI. In addition, PEMS can predict 30-day mortality both in hemodynamically stable and in unstable PE.

Our study is limited by the retrospective data collection, the short follow-up and absence of an external validation of the proposed mortality score. However, it is based on a large patient sample acquired in 2 centers. Clearly, the new score should be validated by prospective, ideally, by multicenter, studies.

In conclusion, a new simple score to predict 30-day mortality in patients with PE based on clinical, radiological, and chemical parameters is proposed. It has an 84.9% sensitivity and 83.0% specificity.
